# Contribution of Nrf2 Modulation to the Mechanism of Action of Analgesic and Anti-inflammatory Drugs in Pre-clinical and Clinical Stages

**DOI:** 10.3389/fphar.2018.01536

**Published:** 2019-01-11

**Authors:** Larissa Staurengo-Ferrari, Stephanie Badaro-Garcia, Miriam S. N. Hohmann, Marília F. Manchope, Tiago H. Zaninelli, Rubia Casagrande, Waldiceu A. Verri

**Affiliations:** ^1^Departamento de Patologia, Centro de Ciências Biológicas, Universidade Estadual de Londrina, Londrina, Brazil; ^2^Departamento de Ciências Farmacêuticas, Centro de Ciências da Saúde, Universidade Estadual de Londrina, Londrina, Brazil

**Keywords:** Nrf2, Keap1, pain, inflammation, oxidative stress, analgesic, anti-inflammatory, antioxidant

## Abstract

Despite the progress that has occurred in recent years in the development of therapies to treat painful and inflammatory diseases, there is still a need for effective and potent analgesics and anti-inflammatory drugs. It has long been known that several types of antioxidants also possess analgesic and anti-inflammatory properties, indicating a strong relationship between inflammation and oxidative stress. Understanding the underlying mechanisms of action of anti-inflammatory and analgesic drugs, as well as essential targets in disease physiopathology, is essential to the development of novel therapeutic strategies. The Nuclear factor-2 erythroid related factor-2 (Nrf2) is a transcription factor that regulates cellular redox status through endogenous antioxidant systems with simultaneous anti-inflammatory activity. This review summarizes the molecular mechanisms and pharmacological actions screened that link analgesic, anti-inflammatory, natural products, and other therapies to Nrf2 as a regulatory system based on emerging evidences from experimental disease models and new clinical trial data.

## Introduction

A cross-talk between varied reactive oxygen species (ROS) and reactive nitrogen species (RNS) is a common feature of inflammatory and painful diseases. These oxidants can be generated by enzymes abundant in immune and non-immune cells (Mittal et al., 2014) as part of protective actions against infections and environmental threats, including microbial or any noxious insults (Janssen-Heininger et al., 2008). The agonist binding to cellular receptors dictates the actions of nicotinamide adenine dinucleotide phosphate (NADPH) oxidases (NOX) and nitric oxide synthases (NOS) to produce superoxide anion and nitric oxide, respectively. Besides the oxidative burst, ROS are also produced in mitochondrial compartments as a result of respiratory chain activity where oxygen consumption is high (Paiva and Bozza, 2014).

Compelling evidence also indicates that oxidative stress is not only related to tissue damage, but also cellular signaling pathways that tightly control cell division, migration and mediator production that ultimately regulate diverse cellular functions (Finkel, 2003; Janssen-Heininger et al., 2008; Pinho-Ribeiro et al., 2016b). Indeed, ROS/RNS sustain their own production and induce the release of cytokines, adhesion molecules, lipid mediators, inflammasome assembly and cyclooxygenase (COX)-2 expression by mechanisms involving the nuclear factor kappa B (NF-κB) activation (Verri et al., 2012; Wardyn et al., 2015; Hennig et al., 2018). NF-κB also induces mitochondrial activity and NADPH oxidase expression in the context of inflammation (Manea et al., 2007; Mauro et al., 2011; Wardyn et al., 2015). Activation of the nuclear factor erythroid 2 (NFE2)-related factor 2 (Nrf2) is central to the disruption of this circle. In fact, *in vivo* studies have shown that Nrf2 signaling has an essential role in limiting neuropathies (Arruri et al., 2017), arthritis (Ferrandiz et al., 2018), colitis (Xu et al., 2018), pneumonia (Athale et al., 2012), pulmonary fibrosis (Yan et al., 2017), skin diseases (Schafer and Werner, 2015), liver (Bae et al., 2013), and kidney damage (Shen et al., 2017), as well as affecting tumor development (Sporn and Liby, 2012). Importantly, the structural features and signaling of Nrf2 protein assign its activity to maintain cellular redox homeostasis (Hayes and Dinkova-Kostova, 2014).

**FIGURE 1:**
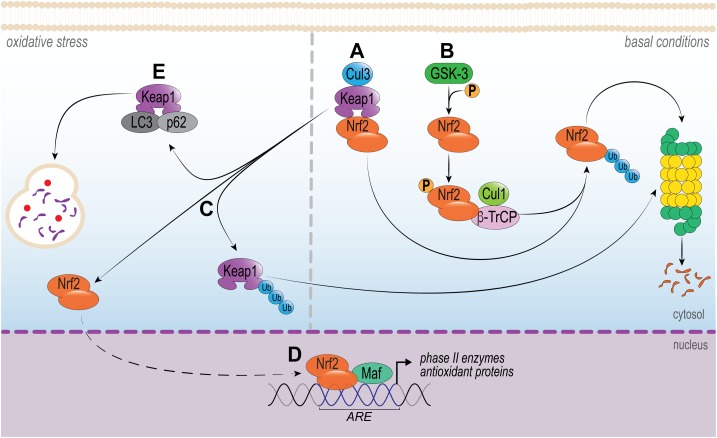
Intracellular signaling pathways that regulate Nrf2. In basal conditions, **(A)** Nrf2 is sequestered in the cytosol by Keap1 by two motifs (ETGE and DLG), which are essential to recruit Nrf2. Keap1 works as a dimeric redox sensitive substrate adaptor for cullin-based E3 ubiquitin ligase, which inhibits the transcriptional activity of Nrf2 via ubiquitination and proteasomal degradation. This signaling is known as canonical pathway. **(B)** Alternatively, Nrf2 is also regulated by a non-canonical pathway. The phosphorylation of Nrf2 by GSK-3 facilitates its recognition by β-TrCP, leading to Cul1-mediated ubiquitination, followed by Nrf2 proteasome degradation. Under oxidative stress or pathological conditions, **(C,D)** Keap1-CUL3 ubiquitin E3 ligase activity decreases and Nrf2 dissociates from Keap1. Nrf2 translocates to the nucleus and heterodimerizes with small musculoaponeurotic fibrosarcoma (Maf) protein and binds to DNA and other transcription partners to setting up a nuclear complex with the ubiquitin-conjugating enzyme UbcM2. These nuclear complexes formed with Nrf2 induce the expression of the ARE-gene battery, such as: NQO1, HMOX1, GCL, GSTs, CAT, SOD, and thioredoxin UDP-glucuronosyltransferase. **(E)** The multifunctional protein p62 and LC3 acts by sequestration of Keap1, which culminates in its autophagic degradation. As a consequence, Nrf2 can translocate to the nucleus and activate ARE.

It is well established that Nrf2 activity is controlled, in part, by the cytosolic protein Kelch-like ECH-associated protein 1 (Keap1), as portrayed in Figure 1. Under homeostatic conditions, Nrf2 levels and its activation are controlled essentially by Keap1. Two Keap1 molecules maintain Nrf2 attached to its DLG and ETH motifs, which favors CUL3-mediated ubiquitination of Nrf2 and subsequent proteasome degradation (Itoh et al., 1999). A small proportion of Nrf2 escapes the inhibitory complex and accumulates in the nucleus to mediate basal antioxidant responsive element (ARE)-dependent gene expression and maintains cellular homeostasis (Kansanen et al., 2013). Conversely, upon oxidative stress or in the presence of electrophilic or activating compounds, the modification of key Keap1 cysteine residues promotes the dissociation of the inhibitory complex and nuclear translocation of Nrf2. In the nucleus, Nrf2 forms a heterodimer with its partner sMAF (v-Maf avian musculoaponeurotic fibrosarcoma oncogene homolog) and binds to ARE, driving the expression of an array of Nrf2-target genes, for example NAD(P)H quinone-oxidoreductase 1 (NQO1), heme-oxygenase 1(HO-1), glutamate-cysteine ligase (GCL), glutathione S-transferases (GSTs), catalase (CAT), superoxide dismutase (SOD) and thioredoxin UDP-glucuronosyltransferase (Nguyen et al., 2009; Ruiz et al., 2013; Hayes and Dinkova-Kostova, 2014). This signaling is defined as the canonical mechanism of Nrf2 pathway (Silva-Islas and Maldonado, 2018). Importantly, this pathway can be modulated by protein kinases involved in signal transduction in the cytosol, such as protein kinase C (PKC), phosphoinositide 3-kinase (PI_3_K) and mitogen-activated protein kinase (MAPK) ERK1/2 (Bloom and Jaiswal, 2003; Nguyen et al., 2003; Manandhar et al., 2007).

Alternatively, the interaction of Keap1-Nrf2 can be disrupted by non-canonical mechanisms (Figure 1). These independent mechanisms involve the disruption of Keap1/Nrf2 interaction by competitive binding of disrupter proteins p62, p53-induced p21 (Toledano, 2009; Best and Sutherland, 2018); DPP3 (Hast et al., 2013), WTX (Karapetian et al., 2005), Prothymosin α (Karapetian et al., 2005), PALB2 (Ma et al., 2012), or BRCA1 (Gorrini et al., 2013) to Keap1 (Lau et al., 2010). Of note, p62 is particularly interesting, since it is the most studied non-canonical pathway of Nrf2 activation. The deficiency in autophagy upregulates p62, that binds to Keap1, thereby inhibiting the Keap1-Cul3-E3 ubiquitin ligase complex and stabilizing Nrf2 (Lau et al., 2010). In addition, PI3K/Akt signaling pathway can activate serine threonine kinase glycogen synthase kinase 3-beta (GSK-3β), which phosphorylates Nrf2 and, in turn, results in the induction of downstream HO-1, glutathione peroxidase, GST A1, NQO-1 and GCL expression (Salazar et al., 2006; Best and Sutherland, 2018). These recent discoveries on the mechanisms of Nrf2 regulation illustrate possible therapeutic strategies to modulate its activity.

Of particular interest is the notion that pharmacological activation of Nrf2 interferes in inflammation (Hayes and Dinkova-Kostova, 2014). It is also important to mention that Nrf2 is expressed in many tissues, mainly those exposed to the environment or associated with detoxification (Itoh et al., 2010). In other words, it is likely that Nrf2 can be exploited as a target in distinct organs. Thus, in this review we will discuss the involvement of Nrf2 in the mechanisms of action of classic analgesic and anti-inflammatory drugs, as well as natural products and other molecules that modulate Nrf2, in the context of experimental models (summarized in Table 1) and human diseases (summarized in Table 2).

**Table 1:** Summary of evidence on analgesic and anti-inflammatory drugs that modulate Nrf2.

Classification	Compound	Disease or experimental model	Dose/Concentration	Outcome	Reference
Opioid analgesics	Morphine	CFA-induced inflammatory pain	*In vivo:* 50 μg/30 μL, i.pl.	Synergy with the induction of Nrf2 to achieve better analgesic effect	Redondo et al., 2017
	Fentanyl	Myocardial I/R injury	*In vivo:* 50 μg/kg, i.v.	Synergy with butorphanol to activate Nrf2-ARE pathway to reduce oxidative stress	Zhang et al., 2016
	[d-Pen(2),d-Pen(5)]-Enkephalin	db/db mice	*In vivo:* 0.15 mg/kg	Synergy with SFN and induction of Nrf2 activation to enhance antinociceptive effect	McDonnell et al., 2017
	SNC-80	db/db mice	*In vivo:*0.5 mg/kg	Synergy with SFN and induction of Nrf2 activation to enhance antinociceptive effect	McDonnell et al., 2017
Non-opioids analgesics	Anandamide	Breast cancer cells	*In vitro:* 2.5 μM	Activation of Nrf2-ARE pathway to induce HO-1 transcription in breast cancer cells	Li H. et al., 2013
	Cannabidiol	LPS-activated BV2 cells	*In vitro:* 10 mM	Activation of Nrf2-Hmox1 and the Nrf2/ATF4 pathways to control LPS-induced activation of microglial cells	Juknat et al., 2013
	Desipramine	Mes23.5 dopaminergic neurons	*In vitro:* 20 μM	Protection of neuronal cell death through Nrf2 activation	Juknat et al., 2013
NSAIDs	Aspirin	Human melanocytes	*In vitro:* 10–90 μM	Protection of human melanocytes against H_2_O_2_-induced oxidative stress via Nrf2 activation	Jian et al., 2016
		Spinal cord contusion model in Sprague-Dawley rats	*In vivo:* 20 mg/kg, i.p.	Suppression of neuronal apoptosis and reduction of inflammation through Nrf2/HO-1 signaling pathway	Wei et al., 2017
	Celecoxibe	Human Umbilical Vein Endothelial Cells	*In vitro:* 1–10 μM	Vascular protection via AMPK-CREB-Nrf2 signaling	Al-Rashed et al., 2018
	Diclofenac	Mosquito fish	*In vivo:* exposure of 1.572 × 10^-3^ μmol – 1.572 μmol	Activation of Nrf2 as a protective mechanism due to large absorption and accumulation of diclofenac	Bao et al., 2017
	Indomethacin	ARPE-19 cells	*In vitro:* 50–250 μM	Inhibition of macrophage infiltration and reduced VEGF levels due to Nrf2 activation	Yoshinaga et al., 2011
	Bromfenac	ARPE-19 cells	*In vitro:* 5–160 μM	Inhibition of macrophage infiltration and reduced VEGF levels due to Nrf2 activation	Yoshinaga et al., 2011
SAIDs	Dexamethasone	Zebrafish larvae	*In vivo:* exposure of 50 nM-50 pM	Nrf2-mediated oxidative stress response	Chen et al., 2017
		Lymphoblastoid cells	*In vitro:*100 nM	Increase of GSH and NADPH levels as well as improved the antioxidant capacity in a Nrf2-dependent manner	Biagiotti et al., 2016
		Human bronchial epithelial cells	*In vitro:* 10^-6^ M	Nrf2/AOX1 pathway enhances airway epithelial barrier integrity	Shintani et al., 2015
	Prednisolone	Zebrafish larvae	*In vivo:* exposure of 50 nM-50 pM	Nrf2-mediated oxidative stress response	Chen et al., 2017
	Triamcinolone	Zebrafish larvae	*In vivo:* exposure of 50 nM-50 pM	Nrf2-mediated oxidative stress response	Chen et al., 2017
	Clobetasol propionate	NSCLC cell lines	*In vivo:* 0.5–1 mg/kg *In vitro:* 10–100 nm	Tumor growth suppression due to high Nrf2 activity	Choi et al., 2017
	Budesonide	Cigarette smoke-, LPS-induced pulmonary	*In vivo:* 1 or 3 mg/kg	Glucocorticoid sensitivity during inflammatory response is dependent on Nrf2-HDAC2 axis	Adenuga et al., 2010
Natural products	Hesperidin-methyl-chalcone	MSU-induced gout arthritis	*In vivo:* 30 mg/kg, p.o.	Inhibition of experimental gout arthritis by decreasing NF-κB activation and inducing Nrf2/HO-1 pathway.	Ruiz-Miyazawa et al., 2018a
	Naringenin	Superoxide anion- induced inflammatory pain	*In vivo:* 50 mg/kg, p.o.	Activation of Nrf2/HO-1 pathway to promote antinociceptive effect	Manchope et al., 2016
		Titanium dioxide (TiO2)-induced chronic arthritis	*In vivo:* 50 mg/kg, p.o.	Activation of Nrf2/HO-1 pathway to promote antinociceptive effect	Manchope et al., 2018
		D-galactose-induced mice brain aging	*In vivo:* 50 mg/kg, p.o.	Nrf2 activation through PI_3_K/Akt pathway	Zhang et al., 2017
	Quercetin	Titanium dioxide (TiO2)-induced chronic arthritis	*In vivo:* 30 mg/kg, i.p.	Inhibition of inflammation in (TiO2)-induced chronic arthritis by decreasing NF-κB activation and inducing Nrf2/HO-1 pathway.	Borghi et al., 2017
		Human normal liver L-02 cells	*In vitro:* 50 μM	Prevention of hepatotoxicity via interacting with Keap1 and blocking the binding of Keap1 with Nrf2	Ji et al., 2015
	Curcumin	Superoxide anion-induced pain-like behavior	*In vivo:* 10 mg/kg, s.c.	Activation of Nrf2/HO-1 pathway to promote antinociceptive effect	Fattori et al., 2015
		Deprivation/ reoxygenation model	*In vivo:* 300 mg/kg, i.p. *In vitro:*5 μM	Protects neurons against ischemic injury through Akt/Nrf2 pathway.	Wu et al., 2013
	Caffeic acid	Acetaminophen-induced liver injury	*In vitro:* human normal liver L-02 cells and HepG2 cells	Protection of APAP-induced hepatotoxicity by inhibiting the binding of Keap1 to Nrf2, and leading to increased expression of HO-1 and NQO1.	Pang et al., 2016
	Vanillic acid	β-amyloid-induced oxidative stress in mice; HT22 cells	*In vivo:* 30mg/kg, i.p. *In vitro:* 100 μM	Neuroprotective effect of against Aβ1-42-induced neurotoxicity through Nrf2 and HO-1 induction	Amin et al., 2017
	Kaurenoic acid	Acute lung injury	*In vivo:* 3 mg/kg, i.t.	Suppression of neutrophilic lung inflammation via Nrf2 activation	Kim et al., 2016
	Glycyrrhizin	Lipopolysaccharide-activated RAW 264.7 cells	*In vivo:* 200 mg/kg, i.p*In vitro:* 2 mM	Reduction of HMGB1 release by induction of p38MAPK/Nrf2/HO-1 signals	Kim et al., 2015
	Rosmarinic acid	Acute liver damage	*In vivo:* 50 mg/kg, p.o.	Hepatoprotective activity due enhanced Nrf2 and HO-1 expression	Domitrovic et al., 2013
		β-amyloid-induced oxidative stress	*In vitro:*1–10 μM	GSK-3β inactivation via the Akt contributing to Fyn dephosphorylation, leading accumulation of Nrf2 in the nucleus	Rong et al., 2018
	Carnosic acid	Ischemia/reperfusion model	*In vivo:* 1 mg/kg, i.p.*In vitro:* 10 mmol/L	Neurons protection from oxidative stress and excitotoxicity through activation of Keap1/Nrf2 transcriptional pathway	Satoh et al., 2008
	Epigallocatechin gallate	Fluoride-induced renal injury	*In vivo:* 40 mg/kg, p.o.	Attenuation of fluoride-induced oxidative stress, renal inflammation and apoptosis by Nrf2 activation	Thangapandiyan and Miltonprabu, 2014
		PM2.5-induced oxidative stress injury	*In vitro:* 50–200 μM EGCG for 24 h	EGCG protects HUVECs from PM2.5-induced oxidative stress injury by upregulating Nrf2/HO-1 via activation of the p38, MAPK and the ERK1/2 signaling pathways	Yang et al., 2015
	Sulforaphane	Nitroglycerin-induced hyperalgesia	*In vivo:* 5 mg/kg, i.p.	Elevated cellular and nuclear levels of the Nrf2 protein	Di et al., 2016
		Spared nerve injury	*In vivo:* 30 mg/kg, i.p.	Decreased Keap1-Nrf2 signaling in mPFC, hippocampus, and muscle contribute to anhedonia susceptibility post-SNI surgery,	Li S. et al., 2018
		COPD alveolar macrophages	*In vitro:* 10 mM	Inhibition of lung inflammation and improvement of bacterial clearance through Nrf2 activation and its downstream target	Harvey et al., 2011
	Capsaicin	HepG2 cells	*In vitro:* 200 μM	Increased production of ROS, Nrf2 activation and induction of HO-1 expression via the PI_3_K/Akt signaling pathways	Joung et al., 2007
Other drugs	Dimethyl fumarate	R6/2 and YAC128 Models of Huntington’s Disease	*In vivo:* 30 mg/kg, p.o.	Increased Nrf2 immunoreactivity in neuronal subpopulations	Ellrichmann et al., 2011
		Chronic experimental autoimmune encephalomyelitis	*In vivo:* 15 mg/kg, p.o.	Reduced macrophage inflammation in the spinal cord and increased levels of IL-10.	Schilling et al., 2006
		Myelin oligodendrocyte glycoprotein induced experimental autoimmune encephalomyelitis	*In vivo:* 15 mg/kg, p.o. *In vitro:* human, rat and mouse astrocytes 100 μM	Increased murine neuronal survival and protected human or rodent astrocytes against oxidative stress. Increased stabilization and activation of Nrf2.	Linker et al., 2011
	Trichostatin A	Inflammatory cystic fibrosis lung disease	*In vitro:* 10 μM	Neff activation and downregulation of innate and adaptive immune responses to reduce lung disease	Bodas et al., 2018
	Sodium butyrate	Permanent middle cerebral artery occlusion	*In vivo:* 5 mg/L	Keap1/Nrf2 dissociation followed by Nrf2 translocation and transcription of HO-1, promoting neuroprotection in stroke	Wang et al., 2012
	Auranofin	U937 and HepG2 cells	*In vitro:* 2.5 μM	Activation of Nrf2/small Maf resulting in transcription of NQO1, GCSh, HO-1 genes and downregulation of inflammatory genes involved in rheumatic diseases	Kataoka et al., 2001; Kim N.H et al., 2010
	15d-PGJ_2_	Ischemia/reperfusion injury	*In vivo:* 0.3 mg/kg, i.v	Prevention of hepatic I/R injury by activation of Nrf2	Kudoh et al., 2014
		Experimental gouty arthritis induced by monosodium urate	*In vivo:* 30 mg/kg, sc	15d-PGJ2-loaded nanocapsules increase mRNA expression of Nrf2/HO-1 signaling and thereby increase in the antioxidant defenses in a PPAR-γ-dependent manner in experimental gout	Ruiz-Miyazawa et al., 2018b
	DHA	Vascular endothelial cell activation by coplanar polychlorinated biphenyls	*In vitro:* 40 μM	Increased DNA binding of Nrf2 and downstream expression of NAD(P)H:quinone oxidoreductase (NQO1), similarly to the Nrf-2 activator sulforaphane.	Majkova et al., 2011
	Resolvin D1	UV radiation-induced skin inflammation	*In vivo:*30 ng/animal i.p.	RvD1 treatment increased the Nrf2 and its downstream targets NQO1 and HO-1 mRNA expression	Saito et al., 2018
	Lipoxin A4	UV radiation-induced skin inflammation	*In vivo:* 10 ng/animal i.p.	Systemic treatment with LXA4 increases mRNA expression and enhanced nuclear factor erythroid 2-related factor 2 (Nrf2) and its downstream target enzyme nicotinamide adenine dinucleotide (phosphate) quinone oxidoreductase (Nqo1) mRNA expression.	Martinez et al., 2018
	DEETGE-CAL-Tat synthetic peptides	Brain injured mice	*In vivo:*15.6 μg/animal i.c.v..	Increase the mRNA levels for Nrf2-driven genes and reduced blood-brain barrier compromise.	Zhao et al., 2011
		Global cerebral ischemia	*In vivo*: arrange of doses, i.c.v. and s.c.	Induced Nrf2 antioxidant/cytoprotective target genes, reduced oxidative stress, and induced strong neuroprotection and marked preservation of hippocampal-dependent cognitive function	Tu et al., 2015
	Head-to-tail cyclic peptide (Peptide 3)	RAW 264.7 cells and LPS (1 μg/mL)	*In vitro*: 1–10 μM	Exhibited anti-inflammatory effects and induced activation of Nrf2-regulated defense system and enhancing the antioxidant capacity.	Lu et al., 2018


**Table 2:** Complete clinical trials related to Nrf2.

Trial registration	Drug	Drug intake	Disease	Enrollment	Study phase	Outcome	Side effects	Country
NCT02023931	Broccoli Sprout Extract	600 μmol systemic delivery or 100 μmol systemic and topical delivery	Healthy subject	10	Early Phase 1	Not provided	Not provided	United States
NCT01335971	Sulforaphane	4.4 and 26.6 mg, daily by mouth	COPD	89	Phase 2	Treatment did not alter the expression of Nrf2 target genes and did not have an effect on levels of other anti-oxidants or markers of inflammation	Nausea (20.69%) Bad taste in mouth (31.03%) Heartburn (24.14%) Bloating/gas (20.69%) Abdominal discomfort (20.69%)	United States
NCT01315665	Broccoli sprouts	100 g of raw broccoli sprouts daily during 5 days	Cystic Fibrosis	15	Not mentioned	Treatment promoted activated Nrf-2 in the cytoplasm of nasal epithelial cells and changes in lymphocyte glutathione levels	Abdominal pain (20%); Back pain (20%); Blood in urine (20%)	United States
NCT01625130	Broccosprouts^®^ (Brassica Protection Products LLC) homogenate	Homogenized with water using a ratio of 1:1.2	Healthy subject	16	Not mentioned	Not provided	Not provided	United States
NCT01715480	Broccosprouts^®^ (Brassica Protection Products LLC) homogenate	Orally daily for 3 weeks	Sickle cell disease	21	Not mentioned	Not provided	Not provided	United States
NCT01845493	Broccosprout homogenate	Orally daily during 3 days	Asthma	16	Phase 1	Not provided	Not provided	United States
NCT02433925	Resveratrol	500 mg per day during 4 weeks	Chronic kidney disease	20	Phase 3	Not provided	Not provided	Brazil
NCT02255422	RTA 408 capsules	Capsules of 2.5, 5, 10, 20, 40, 80, 160 mg, orally	Mitochondrial Myopathy	53	Phase 2	Not provided	Not provided	Denmark United States
NCT02800265	Avmacol	8 tablets every evening for 3 evenings	Healthy subject	10	Not mentioned	Not provided	Not provided	United States
NCT01716858	Sulforaphane-rich Broccoli Sprout Extract	Not mentioned	Schizophrenia	10	Phase 2	Not provided	Not provided	Japan
NCT01269723	Broccoli sprout	Drink the broccoli shake homogenate	Immune Response to Live Attenuated Influenza Virus in Smokers and Non-smokers patients	51	Not applicable	Not provided	Not provided	United States
NCT02592954	Broccoli sprout	500 nM of extract in jojoba oil	Epidermolysis Bullosa Simplex Pachyonychia Congenita	5	Phase 1	Not provided	Not provided	United States
NCT02808624	L-carnosine	500 mg per day	Peripheral Neuropathy on Cancer	65	Phase 1 Phase 2	Not provided	Not provided	Egypt
NCT03115034	Melatonin	6mg per day (3 days before operation to 3 days after operation)	Carotid Endarterectomy	60	Phase 4	Not provided	Not provided	China
NCT02683863	BG00012 (dimethyl fumarate) (Tecfidera^®^)	DMF 120 mg BID for the first 4 weeks of treatment followed by DMF 240 mg BID for 24 weeks	Multiple Sclerosis	20	Phase 4	Not provided	Not provided	United States
NCT03393377	Fluvastatin and Valsartan	Fluvastatin 10 mg and Valsartan 20 mg orally for 30 days	Atherosclerosis	20	Not applicable	Not provided	Not provided	Slovenia
NCT01674231	Grapes in the form of a Freeze-dried Whole Grape Powder	60g freeze-dried whole grape powder with 296 mg polyphenols per day for 4 weeks	Obesity Inflammation Cardiovascular Disease	20	Not mentioned	Treatment enhanced Nrf2 expression in peripheral blood mononuclear cells	Not provided	United States
NCT01802333	Cytarabine Daunorubicin Hydrochloride Idarubicin Vorinostat	Not mentioned	Acute Myeloid Leukemia	756	Phase 3	Not provided	Not provided	Canada United States
NCT01831193	Curcumin	320 mg/day during 8 weeks	Proteinuric Chronic Kidney Disease	120	Phase 3	No effect of CUR was observed on the antioxidant enzymes activities or Nrf2 activation	Not provided	Mexico
UCLA trial	Sulforaphane	25–200 g of broccoli sprout homogenate, daily, during 1–4 days	Healthy smokers	65	Not mentioned	Increased mucosal Phase II enzyme expression in the upper airway of human subjects.	Not provided	United States
NCT00811889	Bardoxolone Methyl	Doses of 25, 75, or 150 mg of Bardoxolone methyl daily, during 24 or 52 weeks	Chronic kidney dis	227	Phase 2	Increased glomerular filtration rate, Effects were maintained for 52 weeks after a 24 weeks administration.	Muscle spasm 42% (25-mg group) 61% (75-mg group) 59% (150-mg group)	United States (Antman et al., 2007)
NCT00529438	Bardoxolone Methyl	Doses of 5, 50, or 100 mg of Bardoxolone methyl daily oral administration during 21 consecutive days of a 28-day cycle for up to 12 cycles.	Advanced Solid Tumors and Lymphomas	47	Phase 1	Increased levels of NQO1 mRNA in PBMCs. Decreased levels of NF-κB and cyclin D1 in tumor biopsies. Increased glomerular filtration rate. Safe with maximum tolerated dose 900 mg/d.	Nausea (>3%) Vomiting (>3%)	United States
NCT01351675	Bardoxolone Methyl	Single dose of 20 mg of Bardoxolone methyl daily	Type 2 diabetes mellitus and stage 4 chronic kidney disease patients	2185	Phase 3	Improved glomerular filtration rate for 24 weeks and persisted at 52 weeks.	Heart failure (6%) Coronary artery disorder (5%)	United States European Union Australia Canada Israel Mexico
NCT01373554	Oltipraz	Doses of 30 or 60 mg of Oltipraz or placebo per oral, twice a day during 24 weeks	Non-alcoholic Fatty Liver Disease	60	Phase 2	Reduced liver fat content and body mass indices. Did not effect insulin resistance, liver enzymes, lipids or cytokines levels	Not provided	Republic of Korea
NCT00956098	Oltipraz	Single dose (30–90 mg) and multiple-dose (60 or 90 mg) of Oltipraz	Liver fibrosis Liver cirrhosis	81	Phase 2	Pharmacokinetics studies shown that oltipraz was rapidly absorbed and demonstrate efficacy and safety	Abdominal discomfort (16%) Dizziness (24%) Dyspepsia (24%)	Republic of Korea


## Modulation of NRF2 by Analgesic Drugs and Synergic Effects

Despite their deleterious side effects, opioids are one of the most effective analgesic drugs employed in the clinic (Knezevic et al., 2017). Opioids produce their pain-relieving actions by interacting with μ, δ or κ opioid receptors (Bovill, 1997). Growing evidence has shown that Nrf2-activators can synergize with opioids to achieve better analgesic effects. Sulforaphane (SFN), for instance, is an activator of Nrf2 transcription factor that enhances the antiallodynic and antihyperalgesic effects produced by morphine. This effect was attributed to local increase in the expression of μ-opioid receptors, as observed in animals with peripheral inflammation. Notwithstanding, SFN enhances the production of HO-1 and NQO1 in the spinal cord and paw tissue, suggesting that the antiallodynic and antihyperalgesic effects of SFN and morphine are produced by a Nrf2 antioxidant-mediated mechanism (Redondo et al., 2017). Fentanyl, another opioid drug, when combined with butorphanol, activates Nrf2-ARE signaling via kappa-opioid receptor. The activation of this pathway increased the expression of downstream genes NQO1 and HO-1 and prevented oxidative stress in myocardial ischemia-reperfusion (I/R) injury model (Zhang et al., 2016). Corroborating these findings, the induction of Nrf2 can also enhance the antinociceptive effects of delta-opioid receptors in diabetic neuropathy associated to type 2 diabetes (McDonnell et al., 2017). Collectively, these data suggest that the use of Nrf2-activators may be beneficial, since lower doses of opioid could be used, thus likely reducing their side effects.

Cannabinoids are molecules that were originally isolated from *Cannabis sativa* and later discovered to be endogenously produced. Cannabinoids represent an alternative pain relief therapy group of drugs with varied origins (Elikkottil et al., 2009). Some molecular effects of cannabinoids depend on Nrf2. One postulated mechanism of action is that endogenous cannabinoids can enhance SOD synthesis and decrease ROS production via Nrf2. These effects were implicated in the neuroprotective effect of cannabinoids in a model of Parkinson’s disease, a progressive nervous system disorder with inflammatory features that affects movement (More and Choi, 2015). Anandamide, an endogenous cannabinoid, leads to Nrf2 activation and downstream HO-1 mRNA expression in MCF-7 and MDA-MB-231 breast cancer cell lines (Li H. et al., 2013). Cannabidiol, a natural cannabinoid constituent isolated from cannabis, controls LPS-induced oxidative stress and inflammation in BV2 cells by inducing Nrf2 and ATF4 transcription factors in a mechanism involving Nrf2-Hmox1 and the Nrf2/ATF4 pathways (Juknat et al., 2013).

Desipramine, a tricyclic antidepressant that displays pain-relief and anti-inflammatory effects, is commonly used in low doses to control neuropathic pain (Roumestan et al., 2007; Hearn et al., 2014). It has been demonstrated that desipramine elevates HO-1 expression through ERK and JNK pathways, leading to Nrf2 activation in Mes23.5 dopaminergic neurons. Desipramine-induced high HO-1 expression also protects dopaminergic neurons from cell death, supporting the notion that this drug could be a promising therapeutic approach to treat neurodegenerative disease (Lin et al., 2012).

In summary, the Keap1/Nrf2/HO-1 axis contributes to the analgesic effects of cannabinoids, anticonvulsant, antidepressant, and enhances the analgesic effects of opioids.

## Modulation of NRF2 by Non-Steroidal Anti-Inflammatory Drugs

Non-steroidal anti-inflammatory drugs (NSAIDs) are a broad class of anti-inflammatory drugs commonly prescribed for pain and inflammation (Cashman, 1996). NSAIDs relieve pain by blocking COX subtypes 1 and 2 enzymes, which results in the inhibition of prostanoid production (Simmons et al., 2004) via peripheral and central actions (Cashman, 1996). Depending on the NSAID, its mechanism of action may also include the inhibition of NFκB activation and induction of pro-resolving lipid mediators (Serhan, 2017). The compounds in willow bark, for example salicylate, provide the basis for aspirin and many other traditional non-selective NSAIDs (Serhan, 2017). Accumulating evidence has shown that various NSAIDs have effects toward the Keap1/Nrf2/ARE pathway (Antman et al., 2007).

Aspirin, the most commonly used NSAID, has free radical scavenging property, specifically the removal of hydrogen peroxide. Aspirin has been reported to have protective effects against H_2_O_2_ in primary human melanocytes by activating Nrf2-ARE pathway and inducing HO-1 expression (Jian et al., 2016). Taken this into account, the authors suggested aspirin as a new antioxidant for the treatment to vitiligo. Aspirin also displayed neuroprotective effects in spinal cord injury model. This effect was attributed to a Nrf2/NQO1/HO-1 signaling pathway-dependent inhibition of neuronal apoptosis, astrocyte activation, oxidative stress and metabolic dysregulation (Wei et al., 2018).

The COX-2 transcription is activated by JNK2/c-jun pathway, which inhibits PI3-K activity and leads to suppression of Nrf2-ARE transcriptional activity (Healy et al., 2005). Corroborating these data, COX-2 selective NSAIDs activate the Nrf2/ARE pathway. Celecoxib is a selective COX-2 inhibitor (Gong et al., 2012) that has been reported to activate AMPK-CREB-Nrf2-dependent signaling and enhance vascular endothelium protection. Treatment of human endothelial cells with celecoxib leads to COX-2 independent signaling via phosphorylation of AMPK, resulting in the nuclear translocation of Nrf2. Together, CREB and Nrf2 pathways upregulate the expression of the antioxidant and anti-inflammatory genes such as HO-1 and H-Ferritin (FHC). In light of this, celecoxib improves endothelial function, minimizing cardiovascular risk in patients (Al-Rashed et al., 2018).

Another group later reported that Mosquito fish (*Gambusia affinis*) exposed to diclofenac for 7 days presented increased expression of *Nrf2* mRNA and its downstream related genes. This response occurred as a protective mechanism due to large absorption and accumulation of diclofenac, which caused the buildup of ROS and induction of the antioxidant responses (Bao et al., 2017). In a model of choroidal neovascularization (CNV), indomethacin or bromfenac showed modulatory activity on Nrf2. In ARPE-19 [human diploid retinal pigment epithelium (RPE)] cells, indomethacin or bromfenac induced the translocation of Nrf2 into the nucleus and high levels of HO-1 in the perinuclear lesion and cytoplasm. *In vivo*, similar therapeutic effects were produced by these NSAIDs. In rat CNV model, indomethacin or bromfenac increased Nrf2 or HO-1 expression, inhibited macrophage infiltration and reduced VEGF levels. These data support NSAIDs treatment as a reasonable therapeutic approach for CNV due to their anti-angiogenic effect and that increasing Nrf2 signaling is a contributing underlying mechanism (Yoshinaga et al., 2011). Overall, these results demonstrate the potential protective effects of a NSAID in inflammatory conditions via the canonical Keap1/Nrf2 pathway.

## Modulation of NRF2 by Steroidal Anti-Inflammatory Drugs (SADs)

Synthetic SADs resemble natural glucocorticoids with peculiar differences in both pharmacodynamics and pharmacokinetics features (Clark and Belvisi, 2012). They possess powerful immunosuppressive actions (Busillo and Cidlowski, 2013) and the most frequently prescribed SADs are prednisone/prednisolone, dexamethasone, and budesonide. Nevertheless, there are several other SADs that are used to treat numerous diseases (Clark and Belvisi, 2012). Synthetic glucocorticoids have been indispensable over the last half-century for treating several inflammatory and autoimmune diseases such as allergies, asthma, rheumatoid arthritis, Graves’ disease, psoriasis, sepsis, and transplanted organ rejections. It is noteworthy that the therapeutic benefits of glucocorticoids are limited by severe adverse effects that develop in patients subjected to long-term use. Adverse effects include osteoporosis, skin atrophy, diabetes, abdominal obesity, glaucoma, growth retardation in children, immunosuppression, inhibition of wound repair, and hypertension hypertension (Miner et al., 2005; Rhen and Cidlowski, 2005).

Regarding the mechanism to achieve the pharmacological effects, glucocorticoids transduce their actions by binding to the glucocorticoid receptor (GR) in the cell cytoplasm. Upon ligand binding, the complex glucocorticoid-GR undergoes conformational change that triggers its translocation to the nucleus. In the nucleus, the glucocorticoid-GR complex binds to glucocorticoid responsive elements, which recruit either coactivator or corepressor proteins. This biding results in the modification of chromatin structure, which can facilitate or inhibit the transcription machinery (Hebbar and Archer, 2003; Nagaich et al., 2004). Moreover, the complex can also interact with other transcription factors such as NF-κB (McKay and Cidlowski, 1999; De Bosscher et al., 2003) and Nrf2 (Ki et al., 2005; Kratschmar et al., 2012). Although glucocorticoids exert their actions mainly through genomic (transactivation and transrepression) mechanisms, non-genomic actions have also been described (Groeneweg et al., 2011).

Experimental approaches based on immunoprecipitation of Nrf2 and its interacting proteins identified GR as a novel Nrf2-binding partner (Alam et al., 2017). In agreement, numerous studies have shown that both natural and synthetic glucocorticoids can modulate Nrf2 activity via GR signaling (Alam et al., 2017; Choi et al., 2017). A microarray analysis of developing zebrafish larvae exposed to glucocorticoids (dexamethasone, prednisolone, and triamcinolone) revealed that Nrf2 was among the top perturbed canonical pathways in oxidative stress response. In agreement with these data, GR signaling can block Nrf2-mediated cytoprotection from oxidative stress (Kratschmar et al., 2012; Alam et al., 2017) and inhibition of GR nuclear translocation restores dexamethasone-induced inhibition of Nrf2/HO-1 expression (Singh and Haldar, 2016). Mechanistically, it was demonstrated that dexamethasone enhances GR recruitment to antioxidant response elements (AREs) without affecting chromatin binding of Nrf2, resulting in the inhibition of acetyltransferase CBP (CREB-binding protein) recruitment and histone acetylation at AREs. This repressive effect was inhibited by the addition of histone deacetylase inhibitors, suggesting that the reduction in Nrf2 transcriptional activation by GR signaling is dependent on the inhibition of histone acetylation (Alam et al., 2017).

In contrast to previous data showing that dexamethasone mitigates Nrf2-mediated response during oxidative stress, in Ataxia telangiectasia lymphoblastoid cells, dexamethasone increased GSH and NADPH levels, as well as improved the antioxidant capacity in a Nrf2-dependent manner. Dexamethasone was shown to induce the translocation of Nrf2 from the cytosol to the nucleus, where its accumulation continued up to 24-h after drug administration to sustain phase II antioxidant gene expression (Biagiotti et al., 2016). In experimental autoimmune encephalomyelitis (EAE), dexamethasone also presented antioxidant effect via up-regulation of Nrf2 and antioxidant enzymes and Nrf2-nuclear translocation (Li B. et al., 2013).

Glucocorticoid-mediated Nrf2 activation also plays an important role in various other physiological and pathological settings. In liver regeneration, Nrf2 has been shown to be a key player (Ishtiaq et al., 2018). A variety of factors regulate hepatic tissue regeneration, among them, augmenter of liver regeneration (ALR) (Dayoub et al., 2013). Chronic release of glucocorticoids increases intracellular ROS levels in hepatocytes, which induces Keap1-Nrf2 conformational changes. These events culminate in the release of the activated Nrf2, an important inducer of ALR expression (Adenuga et al., 2010; Dayoub et al., 2013). In the absence of Nrf2, decreased ALR levels and delayed liver regeneration are observed in mice subjected to partial hepatectomy (Zou et al., 2015). Therefore, glucocorticoid effect on liver regeneration is dependent on Nrf2 regulation of ALR expression. In airway epithelial cells, Nrf2 pathway was identified as essential to steroid (dexamethasone)-induced enhancement of airway epithelial barrier. The transfection of cells with specific siRNA reduced the enhancement of airway epithelial barrier integrity and the accumulation of tight junction and adherent junction proteins at sites of cell–cell contact. Moreover, transfecting cells with aldehyde oxidase 1 (AOX1)-specific siRNA, a downstream enzyme of Nrf2, also reduced steroid-induced enhancement of airway epithelial barrier integrity (Shintani et al., 2015). In cancer cells on the other hand, increased Nrf2 activation due to Keap1 or NRF2 mutations occurs frequently and this seems to be important in the maintenance of these cells. Therefore, Nrf2 inhibition could be a promising therapeutic strategy to target these cells (Ma et al., 2018; Rojo de la Vega et al., 2018). An initial screening of compounds with inhibitory effect on Nrf2 revealed the glucocorticoid clobetasol propionate (CP) as one of the most potent Nrf2 inhibitors. CP prevented nuclear accumulation and promoted Beta-transducin repeats-containing proteins (β-TrCP)-dependent degradation of Nrf2 in a GR- and GSK-3-dependent manner. As a result, CP induced oxidative stress in cancer cells, which strongly suppressed the growth of tumors with Keap1 mutation and high Nrf2 activity (Choi et al., 2017).

In addition to the various effects of GR-signaling via Nrf2, an inverse relation between Nrf2 and glucocorticoid activity has been reported. In a lung inflammation model, it was demonstrated that glucocorticoid activity is regulated by Nrf2. Compared to the WT, Nrf2^-/-^ mice subjected to LPS-induced lung inflammation showed decreased HDAC2 levels and increased markers of inflammation, which was not reversed by the glucocorticoid budesonide (Adenuga et al., 2010). Abnormal lung inflammation and oxidant burden was associated with a significant reduction in HDAC2 abundance and glucocorticoid resistance, thus, glucocorticoid sensitivity during inflammatory response can be dependent on Nrf2-HDAC2 axis.

## Natural Products-Derived Pharmacological Modulators of NRF2

**FIGURE 2:**
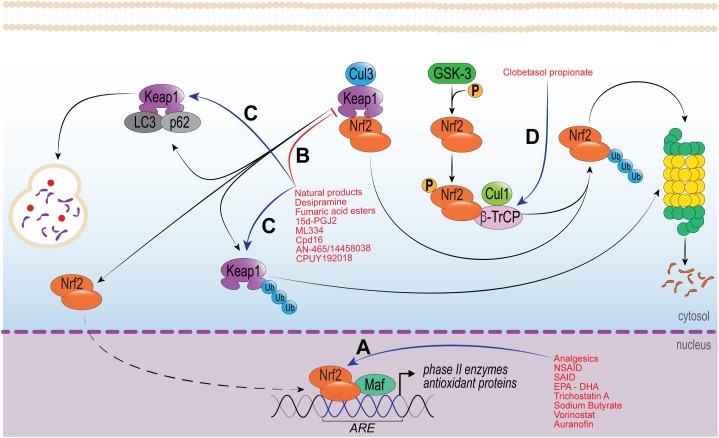
Molecular mechanisms of drugs that modulate Nrf2 activity. **(A)** Group of drugs that increase Nrf2 biding to DNA and/or, Nrf2-Maf affinity to ARE region and/or modulate histone acetyltransferase (HDAC). **(B,C)** Drugs modulating the disruption of Keap1/Nrf2 complex; either **(B)** by protein–protein interaction though Michael addition reaction and/or, **(C)** increasing Keap1 degradation through proteasome or autophagic pathways. **(D)** Drug targeting the degradation of Nrf2 via proteasome by increasing the GSK-3/β-TrCP signaling and reducing Nrf2 activity.

Natural products have been described as extraordinarily rich sources of Nrf2 activators. Phytochemicals are biologically active compounds found in plants and considered to be the main representatives of Nrf2-activators (Kou et al., 2013; Zhu et al., 2016). In fact, analgesic and anti-inflammatory effects produced by many phytochemicals are considered to occur via Nrf2 pathway (Kumar et al., 2014), secondary to increased phase 2 enzymes, GSH production and turnover [GCL and glutathione reductase (GR)], increase of HO-1 and NQO1 levels, and reduced ROS levels (Ma, 2013; Figure 2). It is known that ROS such as superoxide anion induce inflammatory pain (Maioli et al., 2015) via NF-κB activation (Pinho-Ribeiro et al., 2016a) and increased TNFα, IL-1β (Fattori et al., 2015; Yamacita-Borin et al., 2015; Manchope et al., 2016) and endothelin levels (Serafim et al., 2015). These inflammatory peptides sensitize nociceptor sensory neuron terminals that transduce the nociceptive stimuli in peripheral tissue (Pinho-Ribeiro et al., 2017). Thus, the use of phytochemicals to induce Nrf2 pathway and reduce ROS production is a conceivable approach to treat inflammatory and painful conditions.

Nrf2 inducer phytochemicals can be classified into the following distinct classes: Michael acceptors, oxidizable phenols and quinones, isothiocyanates, dithiolethiones, polyenes or vicinal dimercaptans chemical types (Kumar et al., 2014). The common feature between them is the ability to react with sulfhydryl groups by alkylation, oxidation or reduction. Most phytochemicals are Michael acceptors (olefins or acetylenes-conjugated with electron-withdrawing groups) and undergo a conjugate addition with nucleophilic amino acids (cysteine, lysine, and serine) found in electrophile-sensitive proteins (Powers et al., 2002). Moreover, phytochemicals can react with sulfhydryl groups, acting as nucleophiles in Michael reaction signaling, culminating in the up-regulation of phase 2 enzymes (Dinkova-Kostova et al., 2001; Kumar et al., 2014).

Flavonoids are well known for their analgesic and anti-inflammatory properties. Hesperidin-methyl-chalcone (HMC), naringenin and quercetin, for example, exhibit analgesic and anti-inflammatory effects in acute and chronic inflammatory pain conditions. In fact, HMC, naringenin and quercetin inhibited carrageenan-, LPS-, and superoxide anion-induced inflammatory pain by mitigating leukocyte recruitment, oxidative stress, IL-33, TNFα, IL-1β, IL-6 production and NF-κB activation in mice (Maioli et al., 2015; Pinho-Ribeiro et al., 2015, 2016b,c; Manchope et al., 2016). Both the analgesic and anti-inflammatory actions of the aforementioned molecules have been attributed, at least in part, to Nrf2 pathway activation. The flavonoid HMC inhibited gout-induced inflammatory pain reducing synovitis, leukocyte recruitment, oxidative stress, inflammatory cytokines (TNFα, IL-1β, and IL-6) and inducing Nrf2 activation and HO-1 mRNA expression in mice (Ruiz-Miyazawa et al., 2018a). The flavanone naringenin inhibited titanium dioxide/prosthesis-like-induced chronic arthritis by mitigating leukocyte recruitment, cartilage degradation, bone resorption, oxidative stress, and inflammatory cytokine expression (IL-33, TNF-α, IL-1β, and IL-6), and NF-κB activation (Manchope et al., 2018). It is noteworthy that the chronic effect of naringenin in titanium dioxide/prosthesis-like model may be related to its inhibitory effect on superoxide anion-induced inflammatory pain through Nrf2 activation and increased HO-1 mRNA expression (Manchope et al., 2016). Mechanistically, naringenin increased Nrf2/HO-1 through PI_3_K/Akt signaling pathway in D-galactose-induced mice brain aging (Zhang et al., 2017). The flavonoid quercetin has also been reported to inhibit chronic inflammatory pain in titanium dioxide/prosthesis-like-induced chronic arthritis and Ehrlich tumor-induced pain in mice (Calixto-Campos et al., 2015b). The mechanisms attributed to this analgesic effect was reduced leukocyte recruitment, oxidative stress, and inflammatory cytokine production (TNF-α, IL-1β, and IL-6) secondary to increased Nrf2 and phase 2 enzymes HO-1, GPx1 and GR (Calixto-Campos et al., 2015b; Borghi et al., 2017). Quercetin also presents hepatoprotective effect in acetaminophen (APAP)-induced cytotoxicity in human liver L-02 cells by inducing Nrf2 activation and its downstream enzymes HO-1 and GCL. Mechanistically, quercetin-induced Nrf2 activation by Keap1-dependent and Keap1-independent mechanisms. Keap1-dependent mechanism was secondary to the interaction of H-benzene and H-bond of quercetin to Keap-1 Arg415 and Tyr527, and Gly364, respectively. The Keap1-independent mechanism, on the other hand, occurred through JNK MAP kinase signaling pathway. Interestingly, quercetin also induced hepatoprotection by increasing p62 protein via JNK signaling pathway (Ji et al., 2015). Collectively, the flavonoid induction of Nrf2 and its downstream enzymes may be Keap1-dependent and Keap1-independent, and by the non-canonical pathway via p62 (Figure 2). However, further investigations are needed to establish which pathways are being activated by each molecule.

Curcumin, a yellow pigment found in turmeric, inhibited superoxide anion-induced inflammatory pain via the inhibition of leukocyte recruitment, oxidative stress, TNF-α, and IL-1β production and induction of Nrf2 activation and HO-1 expression (Fattori et al., 2015). In addition, this polyphenol inhibited neurological impairment, brain edema and infarction volume, capillary leakage, and oxidative stress in middle cerebral artery occlusion-induced ischemic/reperfusion damage in rats by activating Nrf2 and inhibiting NF-κB (Li et al., 2016). The curcumin induction of Nrf2 expression and its downstream enzymes were dependent of PI_3_K/Akt signaling pathway activation in an *in vitro* model of ischemia/reperfusion (Wu et al., 2013). Studies show that curcumin may also exert therapeutic effect as a Michael acceptor by binding to COX-1, COX-2 (Selvam et al., 2005), and GSK-3β (Bustanji et al., 2009). Moreover, in a randomized double-blind placebo-controlled clinical trial, the positive effect of the dietary supplementation with curcumin on the redox status and Nrf2 activation was observed in patients with non-diabetic or diabetic proteinuria in chronic kidney disease (Jimenez-Osorio et al., 2016).

Cinnamic acid and its derivates are one of the simplest phenolic acids found in nature (Anantharaju et al., 2016). Caffeic acid, a cinnamic acid derivate found in thyme and oregano, possesses analgesic activity. This analgesic effect was observed in the formalin- (second phase) and acetic acid-induced writhing tests, as well as, LPS- and carrageenan-induced mechanical hyperalgesia in rats (Mehrotra et al., 2011). Later studies revealed that caffeic acid also has hepatoprotective and anti-inflammatory effects. In APAP-induced liver injury model, this molecule protected mice by reducing neutrophil recruitment and ROS levels and increasing GSH levels in the liver. Interestingly, caffeic acid alone, i.e., independent of APAP-induced injury, boosted hepatic GSH levels (Pang et al., 2016). These effects were attributed to the induction of Nrf2 and its downstream enzymes HO-1 and NQO1 by caffeic acid, as observed in human liver L-02 cells. Mechanistically, caffeic acid decreased Keap1 content through the H-benzene interaction with Arg415 of Keap1. In addition, the hydrogen atom of the hydroxyl at 3-position in caffeic acid formed water-mediated hydrogen bonds with Ser508, and the carbonyl group of CA could form H-bond with Gly603 and Ser363, enhancing the binding affinity between caffeic acid and Keap1 (Pang et al., 2016).

The flavoring agent vanillic acid is also an analgesic and anti-inflammatory. In carrageenan-induced inflammatory pain model, vanillic acid mitigated hyperalgesia, leukocyte recruitment, oxidative stress, IL-33, TNFα, and IL-1β production, and NF-κB activation in mice. Accordingly, the analgesic and anti-inflammatory effects of vanillic acid were related to Nrf2 activation (Calixto-Campos et al., 2015a). In agreement, vanillic acid also inhibited Aβ1-42-induced oxidative stress, neuroinflammation and cognitive impairment in mice through Nrf2 activation and HO-1 expression (Amin et al., 2017). Although the effects were described to be secondary to Nrf-2 pathway activation, these studies did not show how this occurred. Therefore, further investigations demonstrating the precise molecular mechanism involved in vanillic acid activity are necessary.

Terpenoids are naturally occurring organic chemicals derived from five-carbon isoprene unit comprising mono-, di-, and tri-terpenoids. Kaurenoic acid is a diterpene found in *Sphagneticola trilobata* with analgesic and anti-inflammatory properties *in vivo*. Both carrageenan-induced inflammatory pain and TNF-α and IL-1β production were inhibited by kaurenoic acid in mice (Mizokami et al., 2012). It is postulated that the analgesic effect of this compound could be related to Nrf2 activation, since kaurenoic acid attenuated LPS-induced acute lung injury, neutrophil recruitment and inflammatory cytokine gene expression (TNFα and IL-1β) by activating Nrf2 and regulating the expression of phase 2 enzymes NQO-1, HO-1 and glutamate-cysteine ligase catalytic subunit (GCLC) (Kim et al., 2016). Glycyrrhizin a pentacyclic triterpenoid found in licorice root inhibited CFA-induced mechanical and thermal hyperalgesia in mice paw and increased IL-6, TNFα, and IL-1β levels, microglia activation, HMGB1, and NF-κB activation in spinal cord (Sun et al., 2018). Mechanistically, glycyrrhizin inhibited LPS-induced HMGB1 release through p38/Nrf2-dependent induction of HO-1 in Raw 264.7 cells (Kim et al., 2015). *In silico* molecular docking assay showed that glycyrrhizin interacted directly with 16-mer Nrf2 peptide binding site on Keap1, suggesting that the interference in Keap1/Nrf2 binding was involved (Kamble et al., 2017). These findings suggest that Nrf2 activation by glycyrrhizin may be both Keap1 dependent and independent.

Oxidizable phenols and quinones were one of the first classes described as phase 2 inducers before the discovery of Nrf2/ARE pathway (Prochaska et al., 1985; Kumar et al., 2014). Catechol (1,2-diphenol) and hydroquinone (1,4-diphenol) are Nrf2 inducers that undergo oxidation by cytochrome p450 *in vivo.* This reaction results in the formation of its quinone derivate, which contains a Michael acceptor. This quinone can react with critical cysteine residues in Keap1, resulting in Nrf2 activation and increased phase 2 enzyme expression (Bensasson et al., 2008). Rosmarinic acid is a polyphenol-containing a catechol moiety found in rosemary and peppermint. It has been demonstrated that pre- or post-treatment with this compound mitigates chronic constriction injury (CCI)-induced neuropathic pain by inhibiting mechanical and thermal allodynia, oxidative stress, glial cell activation, and TNFα production (Anantharaju et al., 2016). These effects were related to Nrf2 activation. Rosmarinic acid also inhibited carbon tetrachloride-induced liver intoxication in mice by reducing oxidative and nitrosative stress, TNFα, and COX-2 protein expression, and NF-κB activation, as well as inducing Nrf2 activation and HO-1 in the liver (Domitrovic et al., 2013). Importantly, it was demonstrated that Nrf2 activation ocurred through Akt/GSK-3β/Fyn pathway, because the inhibition of β-amyloid-induced oxidative stress by rosmarinic acid was abrogated by Akt inhibitor LY294002, GSK-3β inhibitor LiCl, Nrf2 shRNA, or Fyn shRNA in PC12 cells (Rong et al., 2018). Nevertheless, it remains to be determined if Nrf2 activation by rosmarinic acid is also dependent on Keap1 interaction. In contrast, the interaction with Keap1 was demonstrated for carsonic acid. This molecule interacts with Keap1 via alkylation of critical cysteine residues. This interaction was central to the inhibition of oxidative stress in immature cortical neurons (Satoh et al., 2008). Moreover, the importance of Nrf2 activation in carsonic acid activity was highlighted by the abrogation of this effect in dominant negative Nrf2 cortical neurons (Satoh et al., 2008). *In vivo*, carsonic acid translocates into the brain, increases the level of reduced glutathione *in vivo*, and protects the brain against middle cerebral artery ischemia/reperfusion (Satoh et al., 2008).

Epigallocatechin gallate (EGCG), a catechin polyphenol found in green tea, inhibited bone cancer-induced pain and neuroinflammation by decreasing TNFα in mice spinal cord (Li and Zhang, 2015). ECCG is protective in fluoride-induced intoxication kidney damage in rats. The mechanism attributed to this effect was decreasing oxidative stress, NFκB activation, and inflammatory cytokine (TNF-α, and IL-6) levels, secondary to Nrf2 activation and upregulation of phase 2 enzymes HO-1, GCL, and GST (Thangapandiyan and Miltonprabu, 2014). Collectively, these data indicate that the analgesic and anti-inflammatory effects described for EGCG could be related to Nrf2 activation. Mechanistically EGCG effects involve the activation of ERK and p38 MAPK signaling pathways. In human umbilical vein endothelial cells, PD98059 (a selective inhibitor of extracellular signal regulated kinase [ERK]-1/2) and SB203580 (a selective inhibitor of p38 MAPK), but not SP600125 [a selective inhibitor of c-jun N-terminal kinase (JNK)], attenuated the EGCG-induced Nrf2 and HO-1 expression. Moreover, silencing Nrf2 abolished EGCG-induced enhancement of cell viability and the upregulation of Nrf2 and HO-1 (Yang et al., 2015). Collectively, the studies discussed above show that oxidizable phenols and quinones also induce Nrf2 and its downstream enzymes through Keap1 dependent and independent mechanisms.

Isothiocyanates are derived from their glucosinolate precursors, which are found in cruciferous plants. Glucosinolates are hydrolyzed by plant enzyme myrosinase or by mammalian gastrointestinal microflora (Fahey et al., 2001; Shapiro et al., 2001) and important biological activity has been attributed to these compounds. Sulforaphane, an isothiocyanate found in broccoli, brussels sprout and cabbage, inhibits CCI-induced neuropathic pain and the expression of inflammatory cytokines, COX-2 and iNOS proteins in the spinal cord. Interestingly, the opioid pathway seemed to be involved in sulforaphane analgesic effect, since naloxone inhibited its anti-allodynic action (Wang and Wang, 2017). Anhedonia (loss of pleasure) is a common feature in patients with neuropathic pain and spared nerve ligation (SNI) in rats, which can induce a depression-like phenotype in some of the animals. This phenotype is related with reduction in Nrf2 levels in medial prefrontal cortex (mPFC), hippocampus, spinal cord and skeletal muscle, but not nucleus accumbens in anhedonia-susceptible compared to anhedonia-resistant and sham rats (Li S. et al., 2018). Sulforaphane pretreatment inhibited SNI-induced mechanical hyperalgesia in anhedonia susceptible and resistant rats and normalized Nrf2 levels in mPFC, hippocampus, spinal cord and skeletal muscle in anhedonia-susceptible rats (Yao et al., 2016). In agreement, sulforaphane also inhibited nitroglycerin-induced hyperalgesia and neuronal activation (c-fos and nNOS immunoreactivity) in trigeminal nucleus caudalis in mice by increasing nuclear Nrf2 and phase 2 proteins HO-1 and NQO-1 in neurons (Di et al., 2016). In other contexts, sulforaphane controls inflammation and improves bacterial clearance in chronic obstructive pulmonary disease (COPD). Mice exposed to cigarette smoke for 6 months and challenged with *Haemophilus influenza* or *Pseudomonas aeruginosa* presented pulmonary inflammation, increased in leukocyte recruitment, and impairment in bacterial clearance by alveolar macrophages. The treatment with sulforaphane ameliorated these events through Nrf2 activation and upregulation of macrophage receptor with collagenous structure (MARCO), its downstream target. In agreement, sulforaphane restored the capacity of bacteria recognition and phagocytosis by alveolar macrophages from COPD patients (Harvey et al., 2011). Mechanistically, sulforaphane seems to induce Nrf2 activation through Keap1 cysteine residue 151 interaction (Hu et al., 2011). Regarding the effects of sulforaphane in humans (Table 2), the oral consumption of sulforaphane doses contained in standardized broccoli sprout homogenate increased phase II enzymes in nasal lavage cells in a placebo-controlled dose escalation trial (Riedl et al., 2009). On the other hand, in a randomized double-blind clinical trial, patients with COPD receiving oral sulforaphane treatment did not present significant changes in Nrf2 target genes or markers of inflammation in alveolar macrophages or bronchial epithelial cells (Wise et al., 2016). Interestingly, although similar doses of sulforaphane were tested, these studies reported different outcomes. Differences in the study subjects may account for this. The first trial was conducted with healthy non-smokers, whereas in the second, the subjects were either active or former smokers with COPD. It is plausible that higher doses of sulforaphane may be necessary to produce significant changes in Nrf2 target genes and disease outcome as the disease severity increases. In this case, inhalable formulation of sulforaphane may be more effective in guaranteeing higher delivery in the lungs. Nevertheless, further studies should be conducted to test this hypothesis.

Capsaicin, a transient receptor potential cation channel subfamily V member 1 (TRPV1) agonist, is a compound found in the red pepper. Acutely, capsaicin induces pain, but chronically, it displays analgesic effects when administrated centrally or peripherally by depleting neuropeptides at supraspinal level (Fattori et al., 2016). Unexpectedly, capsaicin induces the production of ROS, which can interact with NQO1. Subsequently, Nrf2-ARE binding can occur; followed by Nrf2 activation and induction of HO-1 expression via the PI_3_K/Akt signaling pathways in HepG2 cells (Joung et al., 2007). However, it is unknown whether the analgesic effect of chronic capsaicin treatment depends on Nrf2 induction.

In summary, this section of the review brought data from the key phytochemical classes that have analgesic and anti-inflammatory activity through Nrf2 pathway (Figure 2). It is noteworthy that natural products are good candidates to associate with conventional analgesic and anti-inflammatory therapeutic protocols. The association of these molecules could reduce the dose of drugs such as opioids, non-steroidal and steroidal anti-inflammatory, that have several known adverse reactions. Seeking for novel analgesics and anti-inflammatory drugs with lessened side effects is also important and may produce substitute drugs for cases in which the side effects of current drugs do not allow their therapeutic use.

## Other Therapies Modulating NRF2

In addition to the previously discussed classes of drugs, many other compounds have shown important biological activity via Nrf2. There are a variety of Nrf2 inducers, most of which are electrophilic molecules. These molecules react with cysteine thiols of Keap1, being Cys151, Cys273, and Cys288 residues the most prone to electrophile reaction. Electrophile adducts can inhibit Keap1 in two different ways. First, by the induction of a conformational change in Keap1, which results in the loss of its binding capacity to Nrf2. Second, by blocking the interaction between Keap1 and CUL3/RBX1, resulting in sequestration of Keap1 with Nrf2 and further stabilization of newly synthetized Nrf2 (reviewed in Cuadrado et al., 2018; Figure 2).

Fumaric acid esters, including dimethyl fumarate (DMF) and the monoester form monomethyl fumarate (MMF), are the most prominent examples of Keap1 cysteine residue modifiers (Lin et al., 2011). DMF [Tecfidera^®^ by Biogen] is to date the only Food and Drug Administration approved drug registered as NRF2 activator. DMF and other fumaric acid esters have been used to treat psoriasis for over 50 years, when the role of Nrf2 in disease was still unknown. Nevertheless, clinical trials have demonstrated the effectiveness of these compounds in reducing psoriasis area and severity index (Altmeyer et al., 1994), and treating cases of moderate to severe chronic plaque psoriasis (Mrowietz et al., 2017; Tzaneva et al., 2018; Table 2). The mechanisms of action related to these effects include a shift from a T helper (Th)1 toward a Th2 immune response, in addition to the overall decrease in the number of peripheral T cells (Ghoreschi et al., 2011; Tahvili et al., 2015). Considering the immune modulatory actions of fumaric acid esters, it is not surprising that these compounds have also therapeutic effects in other auto-immune diseases such as cutaneous lupus erythematosus (Kuhn et al., 2016; Saracino and Orteu, 2017) and multiple sclerosis (MS) Table 2. DMF was approved in 2013 for the treatment of MS (Xu et al., 2015) and is currently used as the first line treatment of relapsing-remitting MS that does not respond to traditional therapies (Bar-Or et al., 2013). Positive results in EAE, a mouse model for MS, were early indications that fumaric acid esters may have beneficial effects in this disease (Schilling et al., 2006). Schilling et al. (2006) reported important therapeutic effects on the course of disease and histology attributed to the reduction in macrophage-mediated inflammation in the spinal cord and increase in systemic IL-10 levels (Schilling et al., 2006). Accordingly, in this same model of MS, DMF-mediated beneficial effects on clinical course and preservation of myelin, axons, and neurons were observed in WT, but not in *Nrf2*^-/-^ mice (Ellrichmann et al., 2011; Linker et al., 2011). In line with the potent anti-inflammatory effects described, DMF also modulates the immune response in dendritic cells and T cells by reducing the release of inflammatory cytokines (Ockenfels et al., 1998). Moreover, DMF prevents endothelial dysfunction and cardiovascular pathologic ROS formation and inflammation, as well as decrease atherosclerosis and kidney dysfunction in diabetic mice (Tan et al., 2014; Sharma et al., 2017). Overall, the anti-inflammatory and immune modulatory activities of fumaric acid esters indicate that these compounds may also be successful in treating other disease in which chronic inflammation and ROS production are important pathological mechanisms.

As previously discussed in this review, Nrf2 acetylation mediated by histone acetyltransferase/HDAC enhances its transcriptional ability and the expression of downstream targets. This is the mechanism of action of the pan-HDAC inhibitor trichostatin A, which protects against cartilage degradation via the reduction in matrix metalloproteinase (MMP)s and proinflammatory cytokines TNF-α, IL-1β, and IL-6 in osteoarthritis (Cai et al., 2015). Moreover, protective effects via Nrf2 in inflammatory cystic fibrosis lung disease and cerebral ischemic damage have also been observed for trichostatin A (Wang et al., 2012; Bodas et al., 2018). Other HDAC inhibitors, for example sodium butyrate and vorinostat, also mitigate inflammation and the up regulation of MMPs and aggrecanase 2 in human osteoarthritis chondrocytes. As a result, HDAC inhibitors have protective effects against cartilage degradation through mechanisms such as Nrf2 activation and the inhibition of NF-κB and MAPK (Khan and Haqqi, 2018). In addition to the drugs discussed in this section, numerous other Nrf2 inducers acting as electrophilic Keap1 modifiers have been or are currently being tested in clinical trials for the treatment of diseases/conditions such as kidney disease, diabetes, liver diseases (non-alcoholic fatty liver disease, and liver fibrosis and cirrhosis), and cancer. Some examples of these molecules are Bardoxolone methyl (Pergola et al., 2011; Hong et al., 2012; de Zeeuw et al., 2013) and Oltipraz (Kim S.G. et al., 2010; Kim et al., 2017a), which are shown in Table 2.

Auranofin (2,3,4,6-Tetra-O-acetyl-1-thio-beta-D-glucopyranosato-S [triethylphosphine] gold) is a gold(I)-containing antirheumatic drug that possesses anti-inflammatory properties mainly via HO-1 induction. The antirheumatic gold(I)-containing compound selectively activates the DNA binding of the heterodimer Nrf2 and small Maf. Once bound to the ARE or Maf-recognition element, Nrf2/small Maf induces a range of antioxidative stress genes, including *HO-1* and γ-*glutamylcysteine synthetase*, which contribute to the scavenging of ROS and exert anti-inflammatory effects (Kataoka et al., 2001). Moreover, auranofin can elevate cellular levels of Nrf2 by increasing protein stability. Co-immunoprecipitation and Western blot analysis indicated that auranofin inhibits Nrf2 degradation by inducing the dissociation of the Nrf2-Keap1 complex, resulting in nuclear accumulation of Nrf2. Additionally, mechanistic studies revealed that upregulation of Keap1/Nrf2 signaling and downstream HO-1 by auranofin is dependent on Rac1/iNOS induction and MAPK activation (Kim N.H et al., 2010).

The role of lipid mediators in promoting the resolution of inflammation has been widely demonstrated (Serhan et al., 2008). Strikingly, studies are now showing that these effects might be induced via Nrf2 activation. The pro-resolving lipid mediator 15d-PGJ_2_, for example, has been reported to interact with Nrf2. 15d-PGJ_2_ forms an adduct to Keap1 and disrupts Nrf2 ubiquitination, leading to the accumulation of Nrf2 in the nucleus (Joo et al., 2017). Numerous studies have demonstrated the anti-inflammatory activity of 15d-PGJ_2_ via Nrf2 activation (Kudoh et al., 2014; Li et al., 2015; Ruiz-Miyazawa et al., 2018b). In a model of gout arthritis in mice, for example, 15d-PGJ_2_-loaded nanocapsules reduced monosodium urate (MSU)-induced pain, inflammatory cytokine production, and NLRP3 inflammasome and NF-κB activation (Ruiz-Miyazawa et al., 2018b). Treatment with 15d-PGJ_2_-loaded NC mitigated oxidative stress and increased both *Nrf2* and *HO1* mRNA expression, which were reverted by the PPAR-γ inhibitor GW9662. These observations suggest that these effects of 15d-PGJ_2_-loaded NC are PPAR-γ dependent, which is in line with previous studies showing that PPAR-γ activation results in increased Nrf2/HO-1 signaling and antioxidant defenses (Hsu et al., 2013). Further substantiating the central role of Nrf2 in the anti-inflammatory activity of 15d-PGJ_2_, Bretscher et al. (2015) demonstrated that the reduction in IL-6 and IL-12 expression by 15d-PGJ_2_ was not present in Nrf2-deficient myeloid cells (Bretscher et al., 2015). Interestingly, 15d-PGJ_2_ also potentiates macrophage efferocytosis through Nrf2-mediated upregulation of CD36 and HO-1. Macrophage efferocytosis is central to the clearance of apoptotic neutrophils during the resolution of inflammation, thus 15d-PGJ_2_ also promotes the resolution of inflammation (Kim et al., 2017b).

Other lipid mediators that also seem to mediate anti-inflammatory effects via Nrf2 are lipoxin A4 (LXA_4_) and Resolvin D1 (RvD1) (Martinez et al., 2018; Saito et al., 2018). In ultraviolet (UV) radiation-induced skin inflammation in mice, LXA_4_ and RvD1 greatly diminished inflammation, matrix metalloproteinase 9 expression, and sunburn cell counts (Martinez et al., 2018; Saito et al., 2018). These lipids also induced Nrf2 and Nqo1 expression, as well as mitigated oxidative stress. Skin damage induced by UVB irradiation is highly dependent on ROS; and Nrf2 has an important role in the restorative adaptive response to UV radiation-induced inflammation and sunburn reaction (Patwardhan and Bhatt, 2015). Therefore, it is likely that increased Nrf2 expression is important in the protective effect of LXA_4_ and RvD1 against UV radiation-induced skin inflammation. In the same rationale, polyunsaturated fatty acids have also been shown to activate Nrf2 in inflammation. The omega-3 fatty acids docosahexaenoic acid (DHA) and eicosapentaenoic acid (EPA), are sources for the production of Protectin and Resolvin D-series, and Resolvin-E series, respectively (Serhan et al., 2008). Therefore it is conceivable that they also may modulate Nrf2 activity. Both DHA and EPA were shown to be cytoprotective against oxidative insults in endothelial cells in a Nrf2-dependent manner (Lee et al., 2015; Sakai et al., 2017). DHA also significantly mitigated the toxicity and increase in MCP-1 levels by organic pollutants such as polychlorinated biphenyls (PCBs) in vascular endothelial cells; and this effect was attributed to increased Nrf2 DNA binding and downstream expression of antioxidants (Majkova et al., 2011). Although several of these studies have reported Nrf2 pathway as being central to the anti-inflammatory and antioxidant effects of the aforementioned lipid mediators and polyunsaturated fatty acids, the precise molecular mechanism involved in the activation of Nrf2 was not demonstrated. In this sense, the interaction between Nrf2 and these molecules is still unclear.

Although Keap1 oxidation by electrophilic compounds (classic inducers) is essential for Nrf2 activation and its downstream effects (canonical pathway), the lack of selectivity of electrophilic Keap1 inhibitors is frequently overlooked and may account for some of the off-target and undesired side effects. Bardoxolone methyl, for example, can interact with over 500 different proteins, including many different transcription factors (Yore et al., 2011; Zhang, 2013). To overcome the lack of selectivity and off-target effects, a new class of NRF2 inducers that prevent the docking of NRF2 to KEAP1 has emerged (Richardson et al., 2015). The Kelch-DC domain of Keap1 binds to Nrf2 via either its DLG or ETGE motif; both of which are thought to be the major targets of small peptides capable of disrupting Keap1-Nrf2 protein–protein interaction (PPI) (Xu et al., 2015; Gazaryan and Thomas, 2016; Jiang et al., 2016; Saito et al., 2016; Dinkova-Kostova et al., 2017). To date, five families of Keap1-Nrf2 PPI inhibitors have been described: tetrahydroisoquinoline, thiopyrimidine, naphthalene, carbazone, and urea derivatives (reviewed recently in Cuadrado et al., 2018) and studies show that these peptides are excellent candidates to activate Nrf2 due to their potent activity and specificity (Hancock et al., 2012, 2013).

The discovery that the DEETGE sequence in Nrf2 is critical for the Keap1-Nrf2 interaction led to the development of DEETGE-CAL-Tat synthetic peptides. These peptides containing the DEETGE sequence, a caplain cleavage site, and a HIV-Tat cell transduction domain, were shown to disrupt Keap1-Nrf2 interaction and induce Nrf2 genes *in vitro* and *in vivo* in brain injured mice (Zhao et al., 2011). A subsequent study revealed that this peptide also had neuroprotective and cognitive-preserving effects in rats subjected to global cerebral ischemia. The administration of the DEETGE-CAL-Tat peptide strongly enhanced nuclear translocation and DNA binding of Nrf2, as well as expression of known Nrf2-regulated target antioxidant/cell-defense proteins in the hippocampal CA1 region. Intracerebroventricular pre-treatment or peripheral post-treatment also induced robust neuroprotection in the hippocampal CA1 region and strongly preserved cognitive function in this model (Tu et al., 2015). In a study by Lu et al. (2018), on the other hand, the head-to-tail cyclic strategy was applied in the development of novel peptide inhibitors (Lu et al., 2018). A novel cyclic peptide 3 showed high binding affinity with Keap1 and potency in Nrf2 activation at cellular level. This peptide exhibited effective anti-inflammatory effects in mouse RAW 264.7 cells by activating the Nrf2-regulated defense system and enhancing the antioxidant capacity (Lu et al., 2018). Importantly, from the large number of compounds indexed, LH601, benzenesulfonyl- pyrimidone 2, N-phenyl-benzenesulfonamide, and a series of 1,4-diphenyl-1,2,3-triazoles seem to be more promising candidates to inhibit the PPI with KEAP1, due to favorable atomic interaction with KEAP1, affinity, and thermodynamic parameters of binding (Cuadrado et al., 2018). Nevertheless, the effect of these molecules has yet to be tested. Moreover, more studies are necessary to test the effects, potency, and safety and better elucidate the mechanisms of the newly developed small peptides discussed herein.

## Role of Drugs Acting Via NRF2 in Cancer

Chronic inflammation, pain and oxidative stress often accompanies cancer. Nrf2 has been conventionally considered as a tumor suppressor, especially in the early stages of cancer. The definition that Nrf2 is a tumor suppressor comes from its cytoprotective effect against exogenous and endogenous insults such as xenobiotics (Sporn and Liby, 2012). More recently, molecular analysis have stablished that Nrf2 is a oncogenic factor and its activation leads to chemotherapy resistance (Ganan-Gomez et al., 2013). In this context, it is controversial whether the activation of Nrf2 by pharmacological agents with anti-inflammatory and antioxidant properties are useful for the prevention or treatment of cancer.

In experimental studies, the chemopreventive role of Nrf2 inducers was mainly addressed by using the naturally occurring isothiocyanate, sulforaphane. Sulforaphane has received audience because of its ability to simultaneously modulate early stages of carcinogenetic events (initiation) or hamper steps involved in cancer development (Fimognari and Hrelia, 2007; Jiang et al., 2018). Mechanistically, sulforaphane, via Nrf2, promotes DNA protection by inducing phase II enzymes (Morimitsu et al., 2002). This complex association between sulforaphane-Nrf2 is defined by the reversible modification of Keap1 cysteine residues (Hu et al., 2011), interaction with MAPK, phosphatidylinositol 3-kinase (PI_3_K), PKC pathways, NF-κB, or epigenetic modifications (Su et al., 2018). Therefore, the modulation of kinases or DNA methyltransferases results in phosphorylation, nuclear accumulation, and increased transcription and stability of Nrf2. In addition, all effects were observed in nanomolar range (Guan et al., 1990; Su et al., 2018).

Besides sulforaphane, others molecules with distinct mechanisms, including phenethyl isothiocyanate, oltipraz, curcumin, resveratrol, fumaric acid and its esters, and synthetic oleanane triterpenoids also have therapeutic effects in cancer by targeting Nrf2 (Gupta et al., 2004). Of note, although all these chemopreventive molecules can interact with proteins other than Nrf2, indicating that Nrf2-independent mechanisms were also present, their beneficial effects were abrogated or decreased in Nrf2 knockouts (Ramos-Gomez et al., 2001). Additionally, ARE reporter mice and measurements of NAD(P)H:quinone oxidoreductase (*NQO1)* and *GST* transcripts levels were also used to confirm the involvement of Nrf2 (Lewis et al., 2006; Sporn and Liby, 2012). Importantly, all these chemopreventive drugs reduce at low doses the uncontrollable oxidative stress generated by carcinogens that would damage DNA and induce persistent inflammation (Hayes et al., 2010; Hu et al., 2010; Sporn and Liby, 2012). So far, the beneficial role of Nrf2 induction has been widely explored at multiple organ sites including skin (Ben Yehuda Greenwald et al., 2017; Alyoussef and Taha, 2018), lungs (Kumar et al., 2011; To et al., 2015; Creelan et al., 2017; Lin et al., 2017), bladder (Iida et al., 2004; Leone et al., 2017), breast (Pledgie-Tracy et al., 2007; Soto-Balbuena et al., 2018; Soundararajan and Kim, 2018), colon (Rajendran et al., 2015; Guo et al., 2018), pancreas (Kallifatidis et al., 2009), stomach (Fahey et al., 2002), and oral cancer (Bauman et al., 2016; Soundararajan and Kim, 2018).

Because Nrf2 promotes cell survival under stress, it is coherent to assume that an increase in Nrf2 could be protective for cancer cells. In this context, the hyperactivation or unbalanced regulation of Nrf2 may participate in tumor growth or be involved in chemoresistance (Ganan-Gomez et al., 2013). However, only a few studies reporting a cancer-promoting role of Nrf2 have been published.

The frequencies of Nrf2 and Keap1 mutation in tumors are often low (Taguchi et al., 2011). Following the discoveries and characterization, it was observed that common oncogenes, such as *KRAS*, *BRAF*, and *MYC*, increase the transcription and activity of Nrf2, resulting in an increase in cytoprotection and, most notably, a decrease in free radical generation (DeNicola et al., 2011; Sutcliffe et al., 2011). Thus, oncogenes may promote tumorigenesis, in part, in a Nrf2-dependent manner by enhancing the survival of tumor cells (DeNicola et al., 2011). Therefore, considering that cells do not appear to become refractory to repeated activation of the NRF2 pathway by drugs, it is possible that cancer cells utilize a non-mutated pathway to support tumor growth in early stages (Perera and Bardeesy, 2011). Despite these effects, studies point out that increased levels of fumarate, due to chronic exposition to DMF, can become carcinogenic to cells (Sporn and Liby, 2012; To et al., 2015). However, additional studies are required to determine whether phytochemicals, synthetic chemopreventive agents or others drugs targeting Nrf2 increase or decrease cancer risk.

Persistent activation of Nrf2 also enhances resistance to etoposide, doxorubicin, tamoxifen and cisplatin. Alongside with this, many Nrf2 downstream genes, in particular, heme oxygenase-1 (HO-1) have been shown to contribute to the observed Nrf2-dependent chemoresistance (Jozkowicz et al., 2007; Jeddi et al., 2017). In this sense, the abrogation of drug-induced ROS by Nrf2 can confer chemoresistance. Moreover, there is no clear understanding between pharmacological agents acting via Nrf2 and direct resistance, since the persistent activation of Nrf2 is usually a result of a genetic mutation (Homma et al., 2009; Kensler and Wakabayashi, 2010).

**FIGURE 3:**
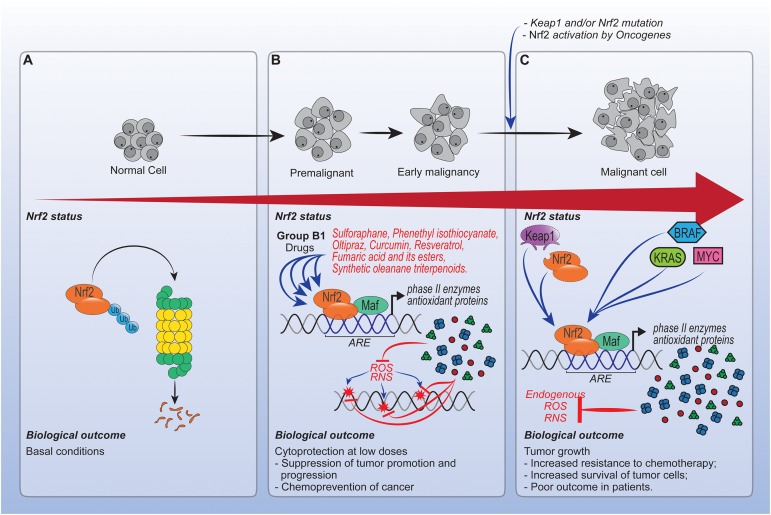
Role of drugs acting via Nrf2 in cancer. **(A)** In normal cells, Nrf2 activity is regulated by canonical and non-canonical pathways, which in the absence of oxidative stress, culminate in Nrf2 proteossomal degradation. **(B)** Enhancing Nrf2 in premalignant and early malignant cells is important to prevent cancer development, specially by low doses of drugs capable of inducing phase II enzymes and antioxidant proteins expression. **(C)** Otherwise, in malignant cells the enhancement of Nrf2 activity caused by mutations such as *Kras*, *Bras* and *Myc*, can protect tumors from the cytotoxic effects of reactive oxygen species (ROS) induced by chemotherapy. However, the effects of drugs that act via Nrf2 at intermediate and chemotherapy stages still need investigation. Overall, the effects of Nrf2 in cancer depend on the biological development stage of tumor cells.

Overall, the timing of Nrf2 activation is important in the context of cancer, as summarized in Figure 3. Enhancing Nrf2 is essential for the prevention of cancer, especially at low doses by drugs that enhance this pathway. On the other hand, in fully malignant cells and in advanced stages of cancer, the enhancement of Nrf2 caused by mutations can protect the tumor microenvironment. At this point, modulating unique redox regulatory mechanism or avoiding use of Nrf2 inducers from general source that greatly accumulate in cells might be effective. However, the direct effects of Nrf2 inducers or even palliative drugs acting via Nrf2, for instance, morphine on cells at intermediate stage of cancer, need further investigation.

## Non-Pharmacological Approaches Targeting KEAP1-NRF2 Pathway

In this section we will discuss data from preclinical studies on non-pharmacological approaches of targeting Keap1-Nrf2 signaling pathway in varied inflammatory disorders. Among the non-pharmacological approaches, the use of interference RNA (iRNA) and genetic constructions targeting the knockdown (KD) of *keap1* gene are widely applied in a variety of *in vivo* and *in vitro* studies. In fact, a *keap1* KD with background on scurfy mice was developed (Suzuki et al., 2017). Scurfy mice are deficient in T_reg_ cells; therefore, they succumb to severe multi-organ inflammation by 4 weeks of age. The systemic activation of Nrf2, by KD of Keap1, considerably ameliorated inflammation and lethality. In addition, increases in Nrf2 activation reduced the number of activated T cells and the amount of pro-inflammatory cytokines (Suzuki et al., 2017).

Similarly, *in vivo* KD of Keap1 decreased fasting-induced steatosis (Xu et al., 2013). The overt-activation of Nrf2 in this particular case decreased the levels of lipid accumulation in the liver and reduced the expression of lipogenic genes and genes related to fatty acid transport. In addition to these findings, the authors propose that Nrf2 plays a role in insulin signaling regulation and enhances insulin sensitivity in skeletal muscle (Xu et al., 2013). The Keap1 KD was also investigated in glomerulosclerosis (Miyazaki et al., 2014). The increase in Nrf2 activity ameliorates podocyte injury caused by an immunotoxin. These findings suggest that the Keap1-Nrf2 system is a promising target in the treatment of chronic liver and kidney diseases.

Although great efforts have been made to study the role of Nrf2 in various diseases, the *in vivo* study of Keap1 is limited to its knockdown, as previously described. Interestingly, Keap1-null mice present postnatal lethality (Wakabayashi et al., 2003). In fact, constitutive activation of Nrf2 culminate in morphological alterations on esophagus and forestomach, manifesting as hyperkeratosis lesions. Two major hypotheses were postulated to explain the lethality of Keap1-null mice. First, a subset of genes for squamous cell differentiation is within the ARE region, therefore, Nrf2 activation induces their expression. Second, the process of desquamation and keratin degradation is dependent on oxidative stress. In this sense, the high amounts of antioxidant proteins prevent keratin oxidation, avoiding desquamation (Wakabayashi et al., 2003). In spite of the protective role Nrf2 plays in many scenarios of diseases, driving its uncontrolled activation may be a dangerous path, as will be discussed further in this section.

The increase o Nrf2 activity was also investigated on human renal tubular HK-2 cell line by KD *keap1* gene with a short hairpin iRNA. Cells with decreased expression of keap1 showed up-regulation in a set of antioxidant and detoxifying proteins, which increased the resistance to cisplatin and doxorubicin cytotoxicity (Jeong et al., 2015). This work demonstrated the potential of Nrf2 to induce the expression of four renal transporters involved in the excretion of drugs, which in a translational manner suggest the importance of Nrf2 in human xenobiotic-induced nephrotoxicity. In a similar way, the KD of *keap1* in Hep2 cancer cells line increased the expression of antioxidant agents and reduced the apoptosis rate when cells were challenged with H_2_O_2_ (Li C. et al., 2018). As discussed in this review, the up-regulation of Nrf2 may favor tumor growth by reducing apoptosis and the relation between Nrf2 and cancer cells is contradictory, therefore, genetic interventions that increase Nrf2 activity may not be a suitable therapeutic approach for cancer.

Despite effective, KD methodologies listed above are limited to the basic research level and obviously not reliable to be considered as therapy for humans today. Nevertheless, the effort of these alternative approaches placed Keap1 as an important target to elucidate the function of genes as well as finding new therapeutic interventions.

## Concluding Remarks

Considering that Nrf2 signaling pathway can regulate at least 600 genes, of which 200 encode cytoprotective proteins that are involved in diseases and the dynamic connections between diseases and drugs, modulating Nrf2 activity is a promising pharmacological approach in inflammatory and painful diseases (Ahmed et al., 2017). Nrf2 activators are commonly naturally occurring and plant-derived, but many others are synthetic compounds as represented by pharmacological classes of analgesics, glucocorticoids, NSAIDs, pro-resolution lipid mediators, electrophilic compounds and others as discussed above. To improve comprehension, we highlighted some examples of drugs with analgesic or anti-inflammatory actions that act via Nrf2 by canonical or non-canonical pathways in Figure 2. Moreover, for a comprehensive overview of the current evidence on molecules that mediate analgesic and anti-inflammatory actions via Nrf2 using *in vivo and in vitro* approaches, we include in Table 1 examples of compounds that belong to the aforementioned pharmacological classes and the main mechanisms demonstrated in experimental models. In Table 2, we provide an overview of molecules tested in completed clinical trials that were discussed in this review. Additionally, we have included molecules that were tested, but no outcome was reported following the completion of the study, for example CXA-10, cytarabine, daunorubicin hydrochloride, fluvastatin, grape powder, idarubicin, L-carnosine, melatonin, resveratrol, RTA 408, valsartan. Data from Table 2 and other clinical trials that were not included herein can be found at: www.clinicaltrials.gov/ct2/results?term=nrf2&Search=Apply&age_v=&gndr=&type=&rslt=.

In conclusion, modulating Nrf2 activity is a promising approach to achieve homeostasis during inflammatory responses where oxidative stress is an essential player. Nevertheless, a better understanding of how the activation or inhibition of Nrf2 can modulate the course and/or outcome of inflammatory diseases is an important strategy for the discovery of new drugs or the repurposing of drugs that target NRF2. Importantly, recent studies focusing on the development of more specific and potent peptides that target Keap1-Nrf2 PPI are promising. However, the effect of many of these molecules have not been tested *in vivo* yet. Therefore, investigations on their effect, mechanisms of action, and safety will be of great value. Further, translational investigations on the therapeutic effect and safety of new or repurposed modulators of Nrf2 pathway in humans is warranted.

## Author Contributions

LS-F, SB-G, MH, MM, TZ, RC, and WV wrote the manuscript, read and approved the final version of the manuscript. All authors contributed equally.

## Conflict of Interest Statement

The authors declare that the research was conducted in the absence of any commercial or financial relationships that could be construed as a potential conflict of interest.
